# OCT findings as a biomarker of disease activity in retinopathy of prematurity

**DOI:** 10.1007/s00417-026-07131-5

**Published:** 2026-02-28

**Authors:** Merve Oral, Hüseyin Baran Özdemi̇r, Şengül Özdek

**Affiliations:** 1 Department of Ophthalmology, School of Medicine, Gazi University, Ankara, 06500 Turkey; 2 Department of Ophthalmology, Turhal State Hospital, Tokat, 60300 Turkey

**Keywords:** ROP, retinopathy of prematurity, Optical coherence tomography, OCT, handheld OCT, Plus disease, Cystoid macular edema, Retinoschisis, Preretinal tissue, Subclinical retinal detachment

## Abstract

**Purpose:**

To determine the value of handheld spectral-domain OCT (SD-OCT) in detecting macular changes in retinopathy of prematurity (ROP) and their association with disease activity.

**Methods:**

This cross-sectional study included 95 eyes of 59 infants diagnosed with treatment-requiring ROP. All eyes underwent indirect ophthalmoscopic examination and handheld SD-OCT imaging prior to treatment. Clinical staging, presence of plus disease, and prior therapies were recorded. OCT images were analyzed for cystoid macular edema (CME), retinoschisis, preretinal tissue, and subclinical retinal detachment. Associations between OCT findings and clinical parameters were evaluated.

**Results:**

Based on indirect ophthalmoscopy, 34.7% of eyes were at stage 3, 34% stage 4 A, 12% stage 4B, 3% aggressive ROP, and 14% presented with persistent avascular retina. CME was detected in 6 eyes (6.3%), significantly associated with plus disease (67%, *p* = 0.04), but not with gestational age, birth weight, or ROP stage. Retinoschisis was identified in 7 eyes (7.3%), predominantly in stage 4 A cases (71.4%). Preretinal tissue (3.1%) was observed mainly in aggressive ROP eyes. OCT refined staging in one case, reclassifying stage 4 A as stage 4B due to macular involvement.

**Conclusion:**

Handheld SD-OCT uncovers macular abnormalities invisible to ophthalmoscopy and can alter disease staging. The significant association of CME with plus disease highlights its potential as biomarkers of disease activity. Routine OCT integration could transform ROP evaluation by refining staging and guiding timely intervention.

## Key Messages

**What is known:**

Handheld OCT reveals macular changes in ROP not detectable with indirect ophthalmoscopy.

**What is known:**

OCT-detected vitreous bands and retinoschisis correlate with advanced disease and traction.

OCT can refine ROP staging by identifying subclinical macular involvement.

This is the first study to demonstrate a significant association of CME with plus disease, indicating a potential biomarker of vascular activity.

Incorporating SD-OCT into routine ROP assessment could enhance diagnostic accuracy, aid in staging, and potentially guide treatment decisions.

## Introduction

Retinopathy of prematurity (ROP) is a vasoproliferative disorder of the immature retina and remains a leading cause of preventable childhood blindness worldwide [1, 2]. Screening and diagnosis rely primarily on binocular indirect ophthalmoscopy, and disease description as well as staging have been standardized and most recently refined by the International Classification of Retinopathy of Prematurity (ICROP)−3 committee [3]. While the peripheral retina is the primary site of pathology in ROP, increasing attention has been directed toward macular changes, which are critical in determining long-term visual outcomes. Accordingly, contemporary clinical decision-making still rests on ophthalmoscopic assessment, while a more granular appraisal of macular architecture may add clinically applicable information in selected cases.

Optical coherence tomography (OCT) provides high-resolution, cross-sectional imaging of retinal microanatomy and has transformed macular diagnostics in many retinal diseases [4]. In neonates, practical barriers—positioning, fixation, and cooperation—historically limited its use; however, the advent of handheld spectral-domain OCT (SD-OCT) has enabled acquisition of clinically useful images in supine position [5]. We believe that with the development of more handy, practical handheld OCT’s with fast image acquisition, OCT may become a standard of care during follow up of ROP. With the increased use of OCT in ROP care, we will have the opportunity to visualize macular and vitreoretinal interface changes that are not readily apparent on clinical examination, potentially augmenting risk stratification and therapeutic planning.

Recent studies utilizing OCT in the evaluation of ROP have reported a range of macular and vitreoretinal abnormalities, including preretinal hyperreflective tissues, vitreous membranes and punctate opacities, epiretinal membranes (ERM), cystoid macular edema (CME), schisis-like changes in the retinal layers, vascular alterations associated with plus disease, and subclinical retinal detachment [6–11]. Collectively, these observations suggest that OCT can reveal subclinical pathology relevant to disease activity and prognosis. Nevertheless, the field still lacks standardized approaches that link specific OCT features to disease stage, plus disease, and treatment decisions in a reproducible manner, and the potential of OCT-derived markers to complement ICROP staging remains incompletely defined.

Therefore, the present study aimed to characterize macular SD-OCT findings across the spectrum of clinically staged ROP and to investigate their relationship with disease severity, including plus/pre-plus status. By examining OCT-detected features such as CME, retinoschisis, preretinal hyperreflective tissue, vitreous membranes, ERM, and macular detachment, we sought to assess their utility as candidate biomarkers of disease activity and to evaluate the incremental value of handheld SD-OCT as an adjunct to conventional examination in the staging and management of ROP.

## Material & methods

This retrospective study included consecutive neonates who underwent handheld SD-OCT imaging at Gazi University Hospital between January 2017 and September 2024.The study adhered to the ethical principles outlined in the Declaration of Helsinki and received approval from the institutional ethics committee. Written informed consent was obtained from the parents or legal guardians of all participants prior to inclusion in the study.

### Imaging protocol

SD-OCT images were obtained prior to laser photocoagulation or vitreoretinal surgery, under general anesthesia. Pupillary dilation was achieved by using topical tropicamide 0.5% (Tropamid, Bilim Pharmaceuticals, Turkey) and phenylephrine 2.5% eye drops. Imaging was performed with a portable, handheld SD-OCT system (Envisu C2300, Bioptigen Inc., Research Triangle Park, NC, USA) while the infants were positioned supine. The SD-OCT acquisition followed an age-specific protocol that adjusted the reference arm position of the device according to estimated axial length and anticipated refractive correction [12]. Scans were centered on the posterior pole, with particular focus on capturing high-resolution scans of the macula. The scanning protocol was adapted from previously published methods [3], with the aim of acquiring the highest-quality macular images. To ensure sufficient data quality and consistency, a minimum of three SD-OCT scans were obtained per patient within 10 min.

Wide-field fundus images were also obtained during the same session with the RetCam 3 system (Clarity Medical Systems, USA). These images were used to assess the ROP stage and zone and were cross-referenced with preoperative indirect ophthalmoscopic findings.

### Image analysis

Following quality control, SD-OCT scans were independently reviewed by two masked graders (H.B.O., M.O.) using proprietary software (InVivoVue v2.4; Leica Microsystems). Eyes with media opacity or poor fixation resulting in suboptimal OCT image quality of the macula were excluded from the analysis. The following features were assessed:

- Vitreous membranes: Linear hyperreflective opacities visible in ≥ 3 consecutive frames, subclassified as tractional (attached to the retinal surface) or non-tractional (parallel but not attached) [7].
- Preretinal hyperreflective tissue: Discrete hyperreflective masses causing posterior shadowing [6].
- ERM: linear hyperreflective structures at the vitreoretinal interface with partial separation from the inner retina [7].
- CME: Intraretinal hyporeflective cystic spaces disrupting retinal lamination [7].
- Retinoschisis: Intraretinal splitting without separation from the retinal pigment epithelium (RPE).
- Macular detachment: neurosensory retinal elevation from the RPE [13].

### Clinical examination

All infants underwent standard ROP examination with binocular indirect ophthalmoscopy. Disease staging was performed according to the International Classification of Retinopathy of Prematurity (ICROP)−3 [3]. For each patient, the following clinical data were recorded: gestational age (GA) at birth, birth weight (BW), ROP stage as determined by indirect ophthalmoscopic examination, and age at the time of surgery or laser photocoagulation.

### Statistical analyses

Statistical analyses were conducted using Microsoft Excel (Microsoft Corporation, Redmond, WA, USA) and the Statistical Package for the Social Sciences (SPSS) version 22.0 (IBM Corp., Armonk, NY, USA). The Shapiro–Wilk test was employed to assess the normality of data distribution. Descriptive statistics were presented as mean ± standard deviation, median, and frequency, as appropriate. For group comparisons, continuous variables were analyzed using the Mann–Whitney U test or Kruskal–Wallis H test, depending on the number of groups. Categorical variables were compared using Fisher’s exact test or the chi-square (χ²) test. Where applicable, Bonferroni correction was applied to adjust for multiple comparisons. Factors associated with OCT findings were first evaluated using univariate analyses, and variables with a p-value ≤ 0.20 were subsequently included in the binary logistic regression model. Results were reported as odds ratios (OR) with 95% confidence intervals (CI), and statistical significance was set at *p* < 0.05.

## Results

A total of 95 eyes from 59 infants (26 girls, 33 boys) who underwent OCT imaging were included in the study. The mean GA at birth was 28.5 ± 3.08 weeks (range: 23–36 weeks), the mean BW was 1317 ± 489 g (range: 550–2800 g) and the mean postmenstrual age (PMA) at the time of SD-OCT imaging was 45.65 ± 8.7 weeks (range: 36–68 weeks). The gender distribution was similar between in the advanced stages (Stage 4 A: 18 eyes in girls and 16 eyes in boys; stage 4B: 6 eyes in girls and 6 eyes in boys).

Based on indirect ophthalmoscopic examination, 33 eyes (34.7%, 23 patients) were classified as stage 3, 3 eyes (3%, 2 patients) had aggressive ROP (A-ROP), 32 eyes (34%, 26 patients) as stage 4 A, 11 eyes (12%, 9 patients) as stage 4B, and 3 eyes (3%, 2 patients) as stage 4 with unspecified macular involvement. In addition, 13 eyes (14%, 9 patients) were stage 0 with persistent avascular retina (PAR) planned for laser photocoagulation. In terms of previous treatments, 30 eyes (31.5%, 20 patients) had received intravitreal anti-VEGF injections for treatment-requiring ROP, and 26 eyes (27.3%, 17 patients) had undergone laser photocoagulation.

SD-OCT imaging revealed a range of macular abnormalities, with retinoschisis and vitreous membranes each identified in 7 eyes (7.3%, in 7 patients), CME in 6 eyes (6.3%, in 5 patients), ERM in 4 eyes (4.2%, in 3 patients), and preretinal hyperreflective tissue in 3 eyes (3.1%, in 2 patients) (Table 1). When correlated with clinical parameters, eyes with pre-plus or plus disease demonstrated a significantly higher prevalence of CME (OR 6.45, 95% CI, 1.05–39.67, *p* = 0.04) (Table 2). Vitreous membranes were significantly more common in stage 4 disease (*p* = 0.01). Additionally, retinoschisis and ERM were more frequently observed in stage 4 A eyes (71.4% and 75%, respectively), although these differences did not reach statistical significance (*p* = 0.5).

**Table 1:** Clinical and OCT characteristics of eyes with OCT findings in our cohort

Patient	Eye	BW	GA	Sex	PMA	ROP stages	Preplus/ Plus	Prior treatment		OCT Findings				
								IV Anti-VEGF	Laser	CME	Retinoschisis	Vitreous band	ERM	Preretinal hyperreflective tissue
1	R	550	25	F	49	A-ROP	+	+	-	-	-	-	-	+
1	L	550	25	F	49	A-ROP	+	+	-	-	-	-	-	+
2	L	1400	32	M	41	3	+	-	-	+	-	-	-	-
2	R	1400	32	M	41	4 A	+	-	+	-	+	-	-	-
3	R	960	27	M	37	3	+	-	-	+	-	-	-	-
3	L	960	27	M	37	3	+	-	-	+	-	-	-	-
4	L	835	26	F	38	3	-	-	-	-	+	-	-	-
4	R	835	26	F	38	4 A	+	-	-	-	+	-	-	-
5	R	1400	31	M	44	3	-	+	+	-	-	-	+	-
6	R	1610	30	F	55	4 A	-	-	+	-	-	-	+	-
7	L	1250	29	F	37	4 A	-	+	+	-	-	-	+	-
7	R	1250	29	F	37	4 A	-	-	+	-	-	+	+	-
8	R	1600	30	M	45	4 A	-	+	+	-	-	-	-	+
9	R	1500	32	F	43	4 A	-	-	-	+	-	-	-	-
10	R	1180	29	M	54	4 A	+	+	+	+	-	-	-	-
11	R	690	24	F	49	4 A	+	+	+	-	+	-	-	-
11	L	690	24	F	49	4 A	+	+	+	-	+	-	-	-
12	L	1129	29	M	59	4 A	-	-	+	+	-	-	-	-
12	R	1129	29	M	59	4 A	-	-	+	-	-	+	-	-
13	L	1200	33	F	39	4B	-	-	-	-	-	+	-	-
14	R	730	26	F	45	4B	+	-	+	-	-	+	-	-
15	L	2050	34	M	67	4B	+	+	-	-	-	+	-	-
16	R	1280	28	F	63	0	-	+	-	-	+	-	-	-
17	L	560	25	F	37	4 A	-	+	-	-	+	+	-	-
18	R	550	25	F	49	A-ROP	+	+	-	-	-	+	-	-

*BW *Birth Weight, *GA *Gestational Age, *PMA *Postmenstrual Age, *ROP *Retinopathy of Prematurity, *IV *Intravitreal, *VEGF *Vascular Endothelial Growth Factor, *OCT *Optical Coherence Tomography, *CME *Cystoid Macular Edema, *ERM *Epiretinal Membrane

**Table 2:** Association between OCT findings and demographic features

	Cystoid Macular Edema			Retinoschisis			Vitreous Membrane		
	Absent (*n*: 89)	Present (*n*: 6)	*p*	Absent (*n*: 88)	Present (*n*: 7)	*p*	Absent (*n*: 88)	Present (*n*: 7)	*p*
GA at Birth* ^¶^	28.49	30.0	0.36	28.77	26.88	0.07	28.56	29.33	0.62
Birth weight* ^¶^	1323	1318	0.37	1327	1254	0.69	1315	1426	0.72
PMA at exam* Gender Female Male	45.74 41 48	44.50 1 5	0.71 0.09	45.60 36 52	46.13 6 1	0.95 **0.04**	45.51 37 51	47.80 5 2	0.79 0.13
Stages ^✢^			0.90			0.31			**0.01**
Stage 0 (PAR)	13	0		12	1		13	0	
A-ROP	3	0		3	0		2	1	
Stage 3	30	3		32	1		33	0	
Stage 4 A	31	3		29	5		31	3	
Stage 4B	12	0		12	0		9	3	
Preplus/Plus$${}^+$$^¶^	**25**	**4**	**0.04**	25	4	0.15	26	3	0.37
Prior Laser Treatment	24	2	0.62	23	3	0.68	23	3	0.34
Prior IV AntiVEGF Treatment${}^+$	29	1	0.43	27	3	0.67	26	4	0.20

*Mann Whitney U test;${}^+$*Fischer exact test* ^¶^*Binary logistic regression*

*GA *Gestational Age, *BW *Birth Weight, *PMA *Postmenstrual Age, *OCT *Optical Coherence Tomography, *ROP *Retinopathy of Prematurity, *PAR *Persistent Avascular Retina, *A-ROP *Aggressive ROP, *IV *Intravitreal, *VEGF *Vascular Endothelial Growth Factor

**Fig. 1:**
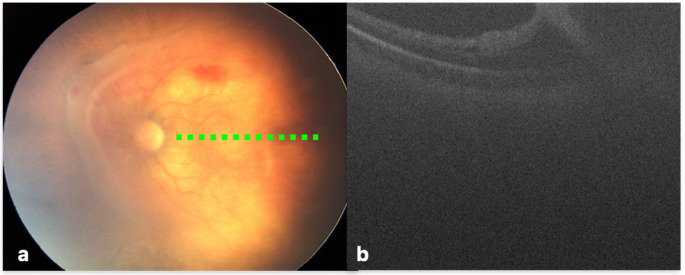
A male infant with a birth weight of 800 g and gestational age of 25-week was referred to our clinic for surgery of the left eye at 37 weeks. In the indirect ophthalmoscopic examination, there was tractional detachment in zone 1 nasally, excluding macula leading to a diagnosis of stage 4 A **(a)**. However, OCT examination showed that a ridge extending to the temporal side of the macula had caused a partial tractional detachment of the macula, which was then re-classified as stage 4B **(b).** The green dashed line marks the approximate location of the OCT B-scans on retina view

Notably, SD-OCT imaging revealed macular detachment, in an eye which was initially classified as stage 4 A based on indirect ophthalmoscopic examination, leading to reclassification of the eye as stage 4B because of macular involvement (Fig. 1).

## Discussion

In this study, we evaluated subclinical macular changes and their associations with clinical findings in infants with different stages of ROP using handheld SD-OCT. Our results demonstrate that OCT provides unique insights beyond indirect ophthalmoscopy, and importantly, reveal novel association between CME and plus disease. 

Vitreous abnormalities on OCT have been variably reported. Lee et al. first described “vitreous material shadowing” without clinical correlation [9], while Zepeda et al. identified vitreous bands in 37% of cases but found no association with GA, BW, PMA, or ROP severity [7]. In contrast, Legocki et al. evaluated tractional bands in relation to plus disease and suggested that they could serve as potential markers of ROP severity [8]. In our study, vitreous bands were less frequent (7.3%) but significantly correlated with advanced stages (4 A–4B, *p* = 0.01), consistent with histological evidence that these bands may reflect organized glial membranes in severe ROP [7]. Although no correlation was observed with other OCT findings, their presence may indicate broader vitreoretinal pathology not fully visualized by current OCT technology.

**Fig. 2:**
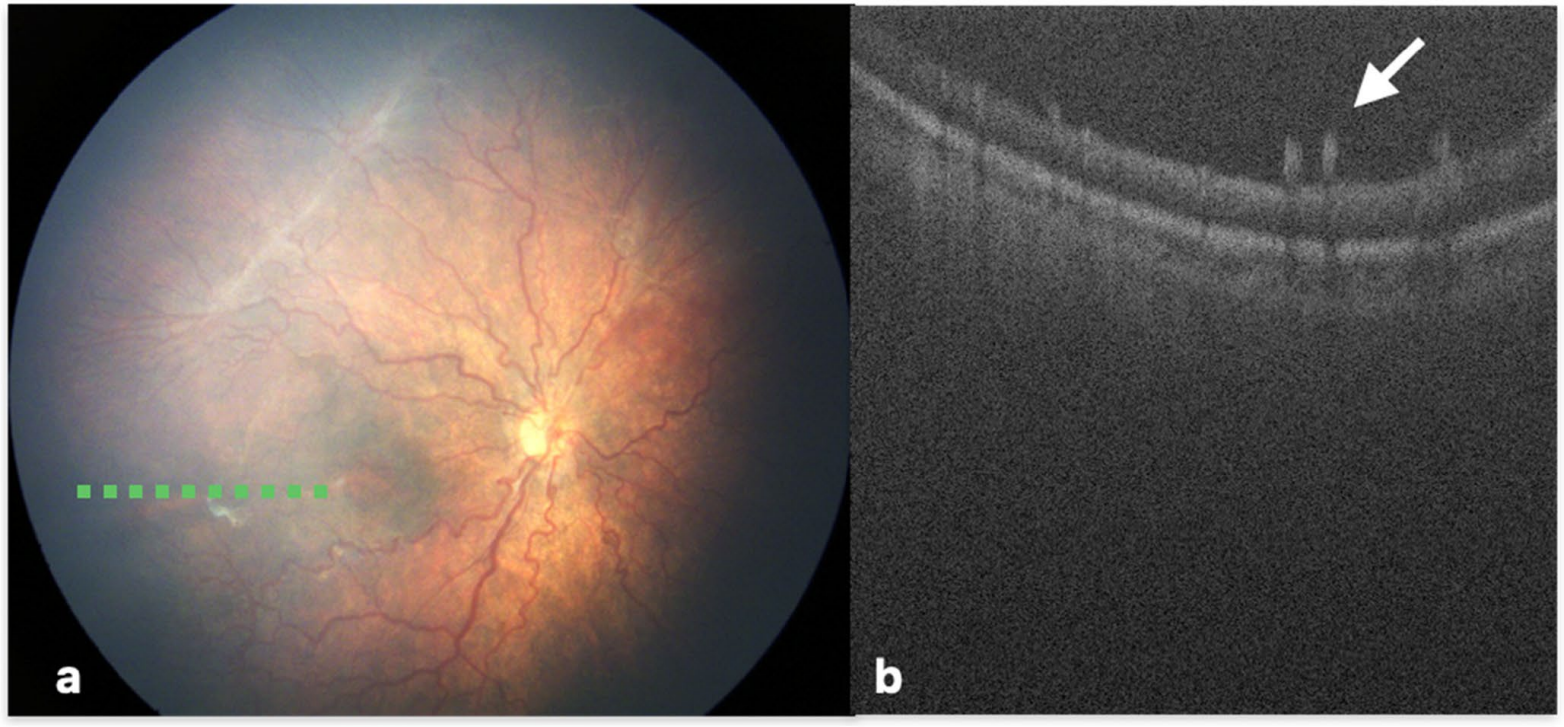
A male infant, born at 25 weeks with a birth weight of 550 g, was scheduled for laser photocoagulation at 49 weeks PMA due to disease progression following intravitreal anti-VEGF injection. Indirect ophthalmoscopy revealed plus disease **(a)**, OCT examination demonstrated the presence of preretinal hyperreflective tissue with posterior shadowing (arrow) **(b)**. The green dashed line marks the approximate location of the OCT B-scans on retina view

Preretinal tissue was first described by Chavala et al. as an isolated extraretinal hyperreflective mass overlying the retina, producing intermittent shadowing artifacts, which indicated a fibrovascular component on OCT [6]. They suggested that these structures may originate from remnants of the hyaloidal vasculature or from abnormal vascular proliferation, as observed particularly in A-ROP. Consistent with this, two of the three cases with preretinal tissue were also diagnosed as A-ROP in our series **(**Fig. 2**)**. Subsequent reports have variably characterized this finding: Lee et al. [9] observed preretinal tissue in 38% of eyes at stage 3 or more advanced disease; Maldonado et al. [14, 15] described it as fibrovascular proliferation resembling “popcorn retinopathy,” noting its location posterior to the ridge and proposing it as a potential marker of progression in zone II disease; in contrast, Xue et al. [16] considered popcorn lesions to be a benign sign of regression. Although the low incidence in our cohort (3.1%) precluded meaningful statistical analysis, the fact that it was encountered in aggressive cases both in the literature and in our study, argues against a benign nature and supports the concept of preretinal tissue as a marker of active or severe disease.

ERM is infrequent in pediatric eyes but has been reported in ROP, often after laser photocoagulation [9] [17]. In our study, ERM was observed in 4.2% of eyes, all of which had received prior laser therapy.

CME remains the most common macular change in premature infants, with reported incidences ranging from 15% to 72% [9, 18, 19]. On OCT, it typically appears as hyporeflective cavities within the inner nuclear layer, often distorting the foveal contour. In our study, CME was observed less frequently (6.3%) than in previous studies, but morphologically consistent with previous descriptions [19]. Its presence has been linked to ROP activity rather than prematurity alone, as highlighted by its absence in infants without ROP [17] and its greater frequency in treatment-requiring stages [18]. Proposed mechanisms include mechanical traction, Müller cell dysfunction, and VEGF-mediated vascular leakage [20–22]. While some studies reported regression following anti-VEGF therapy, paradoxical onset or worsening after anti-VEGF or laser has also been described [20, 23]. Associations with GA or ROP stage remain controversial [19, 22], but in our cohort, CME was significantly associated with plus disease, with an approximately sixfold increased likelihood compared with eyes without plus (OR = 6.45, *p* = 0.04). This finding aligns with evidence linking vascular tortuosity to elevated VEGF levels [24], underscoring CME as a potential marker of increased vascular activity in ROP.

**Fig. 3:**
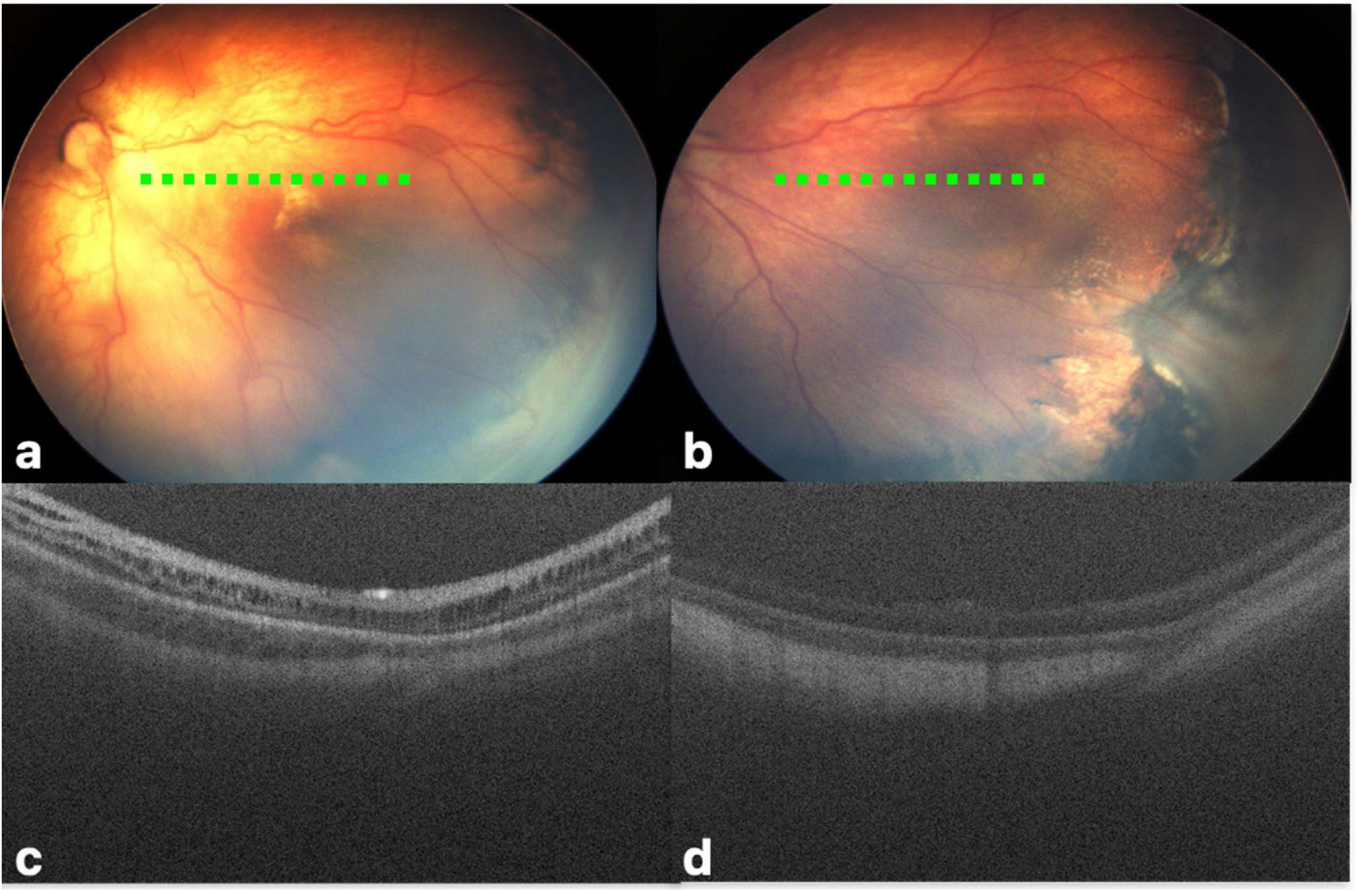
A baby girl with a GA at birth of 24-week was referred to us for surgery at PMA of 49 weeks due to progression following laser photocoagulation **(a**,** b).** An OCT examination revealed retinoschisis, mostly located in the inner layers of the macula **(c)**, which regressed after lens-sparing vitrectomy **(d).** The green dashed line marks the approximate location of the OCT B-scans on retina view

Retinoschisis, defined as separation of retinal layers, has been described in ROP with variable OCT appearances, involving either the inner retina or deeper lamellar layers [6, 10]. Chen et al. observed retinoschisis in 7 of 10 stage 4 A eyes with otherwise normal-appearing macula, proposing an OCT-defined “stage 4A-schisis” to account for poor visual outcomes in advanced ROP [25]. Muni et al. reported retinoschisis was demonstrated in three patients after laser treatment, which was not visible on clinical examination, was presumed to indicate the presence of vitreoretinal traction [26]. In our cohort, stage 4 A ROP was present in the majority (71.4%) of the eyes with retinoschisis and a complete regression of retinoschisis was observed following vitrectomy in some of them **(**Fig. 3). Taken together, these findings suggest that presence of retinoschisis may reflect tractional forces and may serve as an indicator for advanced ROP.

Beyond identifying macular retinoschisis, OCT can also reveal subclinical retinal detachment. Chen et al. reported stage reclassification in several cases, with one eye upgraded from stage 4 A to 4B and two downgraded from 4B to 4 A based on OCT findings [25]. Similarly, in our study, one eye initially classified as stage 4 A on indirect ophthalmoscopy was reclassified as stage 4B after OCT demonstrated macular involvement (Fig. 1). These observations underscore the critical role of OCT in refining disease staging and guiding management decisions in advanced ROP.

This study has several limitations. Foremost is the relatively small sample size, which may affect the statistical power and generalizability of the findings. Additionally, the absence of a control group comprising infants of similar GA and BW without ROP precludes direct comparison and limits the interpretability of the results. Nevertheless, although the OCT findings observed in our cohort are consistent with imaging features previously reported in eyes with ROP, the absence of a control group without ROP remains a limitation, highlighting the need for future prospective studies including appropriate control cohorts. The cohort evaluated use of OCT in eyes with either treatment requiring ROP, or with persistent peripheral avascular retina following anti-VEGF treatment (14% eyes in cohort), thereby constraining the applicability of the findings to earlier stages of the disease. Moreover, the lack of longitudinal OCT follow-up in the majority of cases hinders the ability to accurately determine the timing of onset and resolution of the observed retinal features.

In conclusion, handheld SD-OCT is a valuable adjunctive tool in the evaluation of ROP, offering high-resolution insights into macular architecture that are not detectable through conventional indirect ophthalmoscopy. To our knowledge, this is the first study to demonstrate a significant association of CME with plus disease. These OCT-detected changes may serve as potential biomarkers of disease activity and severity. Incorporating SD-OCT into routine ROP assessment could enhance diagnostic accuracy, aid in staging, and potentially guide treatment decisions. With the development of new OCT machines which can produce wider field of images in a shorter acquisition time, OCT may become the standard of care for ROP patients. Future prospective studies are warranted to validate these findings and to establish the role of OCT biomarkers in routine ROP care.

## Data Availability

No datasets were generated or analysed during the current study.

## References

[1] Gilbert C, Foster A (2001) Childhood blindness in the context of VISION 2020–the right to sight. Bull World Health Organ 79(3):227–232 11285667 PMC2566382

[2] Fierson WM (2018) Screening examination of premature infants for retinopathy of prematurity. Pediatrics. 10.1542/peds.2018-3061 30478242 10.1542/peds.2018-3061

[3] Chiang MF, Quinn GE, Fielder AR, Ostmo SR, Paul Chan RV, Berrocal A, Binenbaum G, Blair M, Peter Campbell J, Capone A Jr et al (2021) International classification of retinopathy of Prematurity, third edition. Ophthalmology 128(10):e51–e68 34247850 10.1016/j.ophtha.2021.05.031 PMC10979521

[4] Huang D, Swanson EA, Lin CP, Schuman JS, Stinson WG, Chang W, Hee MR, Flotte T, Gregory K, Puliafito CA et al (1991) Optical coherence tomography. Science 254(5035):1178–1181 1957169 10.1126/science.1957169 PMC4638169

[5] Scott AW, Farsiu S, Enyedi LB, Wallace DK, Toth CA (2009) Imaging the infant retina with a hand-held spectral-domain optical coherence tomography device. Am J Ophthalmol 147(2):364–373e362 18848317 10.1016/j.ajo.2008.08.010

[6] Chavala SH, Farsiu S, Maldonado R, Wallace DK, Freedman SF, Toth CA (2009) Insights into advanced retinopathy of prematurity using handheld spectral domain optical coherence tomography imaging. Ophthalmology 116(12):2448–2456 19766317 10.1016/j.ophtha.2009.06.003 PMC3514074

[7] Zepeda EM, Shariff A, Gillette TB, Grant L, Ding L, Tarczy-Hornoch K, Cabrera MT (2018) Vitreous bands identified by handheld Spectral-Domain optical coherence tomography among premature infants. JAMA Ophthalmol 136(7):753–758 29799932 10.1001/jamaophthalmol.2018.1509 PMC6136047

[8] Legocki AT, Zepeda EM, Gillette TB, Grant LE, Shariff A, Touch P, Lee AY, Ding L, Estrada MM, Tarczy-Hornoch K et al (2020) Vitreous findings by handheld Spectral-Domain OCT correlate with retinopathy of prematurity severity. Ophthalmol Retina 4(10):1008–1015 32446843 10.1016/j.oret.2020.03.027 PMC7541565

[9] Lee AC, Maldonado RS, Sarin N, O’Connell RV, Wallace DK, Freedman SF, Cotten M, Toth CA (2011) Macular features from spectral-domain optical coherence tomography as an adjunct to indirect ophthalmoscopy in retinopathy of prematurity. Retina 31(8):1470–1482 21792089 10.1097/IAE.0b013e31821dfa6d PMC3165115

[10] Joshi MM, Ciaccia S, Trese MT, Capone A Jr. (2006) Posterior hyaloid contracture in pediatric vitreoretinopathies. Retina 26(7 Suppl):S38–41 16946676 10.1097/01.iae.0000244287.63757.5a

[11] Maldonado RS, Yuan E, Tran-Viet D, Rothman AL, Tong AY, Wallace DK, Freedman SF, Toth CA (2014) Three-dimensional assessment of vascular and perivascular characteristics in subjects with retinopathy of prematurity. Ophthalmology 121(6):1289–1296 24461542 10.1016/j.ophtha.2013.12.004 PMC4070381

[12] Maldonado RS, Izatt JA, Sarin N, Wallace DK, Freedman S, Cotten CM, Toth CA (2010) Optimizing hand-held spectral domain optical coherence tomography imaging for neonates, infants, and children. Invest Ophthalmol Vis Sci 51(5):2678–2685 20071674 10.1167/iovs.09-4403 PMC2868489

[13] Joshi MM, Trese MT, Capone A Jr. (2006) Optical coherence tomography findings in stage 4A retinopathy of prematurity: a theory for visual variability. Ophthalmology 113(4):657–660 16581425 10.1016/j.ophtha.2006.01.007

[14] Maldonado RS, Toth CA (2013) Optical coherence tomography in retinopathy of prematurity: looking beyond the vessels. Clin Perinatol 40(2):271–296 23719310 10.1016/j.clp.2013.02.007 PMC3947541

[15] Wallace DK, Kylstra JA, Greenman DB, Freedman SF (1998) Significance of isolated neovascular tufts (popcorn) in retinopathy of prematurity. J AAPOS 2(1):52–56 10532368 10.1016/s1091-8531(98)90111-2

[16] Xue K, Huang X, Xu S, Zhang T, Wang X, Zhang M, Ruan L, Ni Y (2020) The evolution of isolated neovascular tufts (popcorn) in retinopathy of prematurity. Retina 40(7):1353–1358 31181037 10.1097/IAE.0000000000002596

[17] Gursoy H, Bilgec MD, Erol N, Basmak H, Colak E (2016) The macular findings on spectral-domain optical coherence tomography in premature infants with or without retinopathy of prematurity. Int Ophthalmol 36(4):591–600 26750097 10.1007/s10792-016-0176-9

[18] Vinekar A, Avadhani K, Sivakumar M, Mahendradas P, Kurian M, Braganza S, Shetty R, Shetty BK (2011) Understanding clinically undetected macular changes in early retinopathy of prematurity on spectral domain optical coherence tomography. Invest Ophthalmol Vis Sci 52(8):5183–5188 21551410 10.1167/iovs.10-7155

[19] Maldonado RS, O’Connell R, Ascher SB, Sarin N, Freedman SF, Wallace DK, Chiu SJ, Farsiu S, Cotten M, Toth CA (2012) Spectral-domain optical coherence tomographic assessment of severity of cystoid macular edema in retinopathy of prematurity. Arch Ophthalmol 130(5):569–578 22232366 10.1001/archopthalmol.2011.1846 PMC3515869

[20] Dubis AM, Subramaniam CD, Godara P, Carroll J, Costakos DM (2013) Subclinical macular findings in infants screened for retinopathy of prematurity with spectral-domain optical coherence tomography. Ophthalmology 120(8):1665–1671 23672969 10.1016/j.ophtha.2013.01.028 PMC3737379

[21] Gariano RF (2010) Special features of human retinal angiogenesis. Eye 24(3):401–407 20075971 10.1038/eye.2009.324

[22] Anwar S, Nath M, Gottlob I, Proudlock FA (2023) Severity of cystoid macular oedema in preterm infants observed using hand-held spectral domain optical coherence tomography improves weekly with postmenstrual age. Eye (Lond) 37(14):3009–3014 36928228 10.1038/s41433-023-02461-8 PMC10516860

[23] Erol MK, Coban DT, Özdemir Ö, Tunay Z, Bilgin AB, Dogan B (2015) Spectral-domain OCT analyses of macular changes after Ranibizumab therapy for type 1 retinopathy of prematurity. J Pediatr Ophthalmol Strabismus 52(3):152–158 25859685 10.3928/01913913-20150326-12

[24] Liang T, Qian Z, Tao Y, Peng Y, Cui Y, Zhang C, Peng C, Liu L, Hu M, Li L et al (2022) The relationship between the aqueous VEGF level and the severity of type 1 retinopathy of prematurity. J Clin Med. 10.3390/jcm11185361 36143009 10.3390/jcm11185361 PMC9501342

[25] Chen X, Prakalapakorn SG, Freedman SF, Vajzovic L, Toth CA (2020) Differentiating retinal detachment and retinoschisis using handheld optical coherence tomography in stage 4 retinopathy of prematurity. JAMA Ophthalmol 138(1):81–85 31774474 10.1001/jamaophthalmol.2019.4796 PMC6902125

[26] Muni RH, Kohly RP, Charonis AC, Lee TC (2010) Retinoschisis detected with handheld spectral-domain optical coherence tomography in neonates with advanced retinopathy of prematurity. Arch Ophthalmol 128(1):57–62 20065217 10.1001/archophthalmol.2009.361

